# The concomitant effects of self-limiting insect releases and behavioural interference on patterns of coexistence and exclusion of competing mosquitoes

**DOI:** 10.1098/rspb.2021.0714

**Published:** 2021-05-26

**Authors:** Maisie Vollans, Michael B. Bonsall

**Affiliations:** Mathematical Ecology Research Group, Department of Zoology, University of Oxford, Oxford OX1 3PS, UK

**Keywords:** reproductive interference, behavioural ecology, vector control, disease ecology, release of insects carrying a dominant lethal, sterile insect technique

## Abstract

*Aedes aegypti* is the dominant vector of dengue, a potentially fatal virus whose incidence has increased eightfold in the last two decades. As dengue has no widely available vaccine, vector control is key to reducing the global public health burden. A promising method is the release of self-limiting *Ae. aegypti*, which mate with wild *Ae. aegypti* and produce non-viable offspring. The resultant decrease in *Ae. aegypti* population size may impact coexistence with *Ae. albopictus*, another vector of dengue. A behavioural mechanism influencing coexistence between these species is reproductive interference, where incomplete species recognition results in heterospecifics engaging in mating activities. We develop a theoretical framework to investigate the interaction between self-limiting *Ae. aegypti* releases and reproductive interference between *Ae. aegypti* and *Ae. albopictus* on patterns of coexistence. In the absence of self-limiting *Ae. aegypti* release, coexistence can occur when the strength of reproductive interference experienced by both species is low. Results show that substantial overflooding with self-limiting *Ae. aegypti* prevents coexistence. For lower release ratios, as the release ratio increases, coexistence can occur when the strength of reproductive interference is increasingly high for *Ae. albopictus* and increasingly low for *Ae. aegypti*. This emphasizes the importance of including behavioural ecological processes into population models to evaluate the efficacy of vector control.

## 1. Introduction

Vector-borne diseases account for 17% of all infectious diseases and cause more than 700 000 deaths annually [1]. For example, dengue, a potentially fatal virus spread by *Aedes* mosquitoes, has increased eightfold in incidence over the last two decades [2]. Coupled with the rise in average global temperatures, the total burden on public health caused by vector-borne diseases is likely to further increase: Messina *et al.* [3] predicted 2.25 billion more people will be at risk of dengue by 2080 compared to 2015. In areas where vector-borne diseases are already present, pathogen replication, vector survival, reproduction, biting rate and the length of transmission seasons are set to increase with environmental change [3–5]. Furthermore, the global distribution of vectors is likely to widen: for instance, the global abundance of *Aedes aegypti* is predicted to increase by 20% or 30% by the end of the century, for a low and high carbon dioxide emission future, respectively [6]. Thus, it is increasingly important to have robust strategies to manage and control vector-borne diseases. As many vector-borne diseases have no widely available vaccine or disease-specific drugs (e.g. [6–8]), the key is employing methods to control vector population size.

However, the environment and human health can be negatively impacted by conventional, chemical-based vector control methods, such as the mass spraying of insecticide. Even pyrethroids, which have a low toxicity compared to many other insecticides [9], decrease the diversity of non-target small-bodied arthropods [10,11], cause aquatic toxicity [12,13] and negatively impact the human male reproductive system [14,15]. Additionally, the efficacy of chemical control is declining as insecticide resistance spreads [16]. Therefore, there is a growing imperative to develop novel approaches for controlling disease vectors. As such there has been a continued focus on the use of self-limiting insects for the control of mosquito-borne diseases [17–19]; a technique with substantial benefits [18], which is less costly than alternative control methods [19].

Self-limiting insects decrease the number of offspring contributing to the next generation by competing with wild insects for mates and subsequently producing non-viable offspring. Self-limiting systems include the sterile insect technique (SIT), where insects are irradiated so that they cannot produce viable offspring [20], and the release of insects carrying a dominant lethal (RIDL), where insects are genetically engineered to be homozygous for a dominant lethal genetic construct [17,18]. These methods target single species that are detrimental to human well-being, and do not require the direct or indirect release of harmful chemicals, and thus are considered relatively environmentally benign [19]. Previous use of SIT has been successful—resistance to the technique is infrequent, and it has successfully eliminated and controlled multiple insect pests (see Alphey *et al*. [20]).

However, by altering the population size of the target species, the release of self-limiting insects may have indirect environmental impacts on wider biodiversity—for instance, by affecting the interspecific interactions of the target species [21]. These indirect effects could be substantial when the population size of the heterospecific species is highly coupled to the population size of the target species. This is true for species that have a similar ecological niche to the target species, and are therefore likely to compete strongly with it for resources.

*Aedes aegypti* and *Aedes albopictus*, the two principal vectors of dengue, have overlapping realized ecological niches: they are both anthropophilic, have similar diurnal peak activity periods, use hosts to find mates, exist in urban and suburban settings and deposit their eggs above the water line in (often ephemeral) natural and artificial pools of water [22–28]. However, the two species cannot reproduce to form viable offspring [29]. Despite these species being native to different continents, their range expansion has resulted in frequent overlap of spatial distribution, causing either exclusion, or coexistence [30].

Two key mechanisms influencing coexistence patterns between *Ae. aegypti* and *Ae. albopictus* are interspecific resource competition and reproductive interference (reviewed by Lounibos & Juliano, [31]). Reproductive interference is where incomplete species recognition results in heterospecifics engaging in mating activities which do not produce viable offspring and cause a fitness cost to one or both of the species involved [31,32]. Thus, reproductive interference is distinct from competition, as the fitness costs incurred are not due to shared limited resources [33]. Instead, they can occur directly by female harassment and reduction in fertility, and indirectly through wasted courtship/handling time and energy [31,33]. Reproductive interference occurs across a wide range of taxa, as summarized by Gröning & Hochkirch [31]. Where two species coexist, theoretical studies have shown reproductive interference is more likely to cause exclusion than interspecific resource competition [34]. When both are considered together, reproductive interference acts synergistically with resource competition to promote exclusion; their combined effect is greater than the sum of their independent effects [35].

Reproductive interference occurs between *Ae. aegypti* and *Ae. albopictus* both in the field [36], and in the laboratory [37,38]. As discussed by Paton & Bonsall [33], at least four types of reproductive interference [31] occur between *Aedes* species: misdirected courtship, heterospecific mating attempts, erroneous female choice and heterospecific mating [36–38]. While the impact of reproductive interference upon the coupled population dynamics of *Ae. aegypti* and *Ae. albopoictus* has previously been analysed [33,35], the consequences of this mating disruption and behavioural interference on patterns of coexistence have not been explored in combination with the release of self-limiting *Ae. aegypti*.

*Ae. aegypti* is a key target for self-limiting techniques, as the dominant vector of dengue, and a vector of chikungunya, yellow fever and Zika viruses [1]. In regions where *Ae. aegypti* and *Ae. albopictus* coexist, their population dynamics are strongly coupled. As *Ae. albopictus* is a secondary vector to dengue, and carrier of chikungunya, yellow fever and Zika viruses, it is epidemiologically relevant: assessments of the impact of the release of self-limiting *Ae. aegypti* need to consider the impact upon *Ae. albopictus* populations.

Here, we form a theoretical framework (based on modified ecological competition equations) to investigate the interaction between the release of self-limiting *Ae. aegypti* and the impact of reproductive interference between *Ae. aegypti* and *Ae. albopictus* populations on patterns of coexistence and exclusion. In order to disentangle the effects of reproductive interference caused by wild mosquitoes and self-limiting *Ae. aegypti*, we examine scenarios where the self-limiting *Ae. aegypti* reproductively interfere with *Ae. albopictus*, and where they do not. Our results inform on the developing pragmatic applications and policy developments around the ecological (and epidemiological) consequences of the release of self-limiting *Ae. aegypti* to manage the dengue disease burden.

## 2. Methods

**Figure 1.**
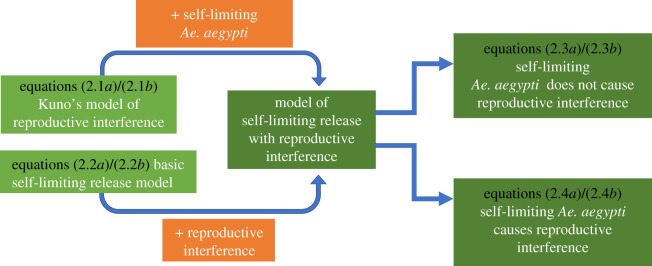
Summary of the development of the models of self-limiting *Ae. aegypti* release with reproductive interference from the baseline models. (Online version in colour.)

We used a set of simple differential equation models to assess how the release of self-limiting *Ae. aegypti* impacts its coexistence with *Ae. albopictus* when there is reproductive interference. Models are summarized in figure 1.

We use two baseline models: Kuno's model of reproductive interference ([34], equations (2.1*a*) and (2.1*b*)) and a basic model of self-limiting *Ae. aegypti* release (equations (2.2*a*) and (2.2*b*)). The former examines the impact of reproductive interference in the absence of self-limiting *Ae. aegypti* release, while the latter explores the effect of self-limiting *Ae. aegypti* release without reproductive interference. The further two models build on these baseline models, to assess the combined impact of self-limiting *Ae. aegypti* release and reproductive interference: first when the self-limiting *Ae. aegypti* do not reproductively interfere with *Ae. albopictus* (equations (2.3*a*) and (2.3*b*)) and, second, when they do (equations (2.4*a*) and (2.4*b*)). Comparisons between these models allowed the impact of self-limiting *Ae. aegypti* release, and reproductive interference on coexistence to be examined separately and in combination. For all models with self-limiting *Ae. aegypti* release, we used a proportional release policy [39]: at each time point, the number self-limiting *Ae. aegypti* released is proportional to the number to wild *Ae. aegypti*.

As with our previous work [33] other demographic processes such as birth and death rate [40,41] were assumed to be equal for both *Aedes* species and parameters were varied to allow the results to be investigated for a variety of environmental contexts [42,43], table 1.

**Table 1.** Canonical parameter values for *Aedes* mosquitoes. The values of subscripted parameters were varied between *Ae. aegypti* and *Ae. albopictus*. The relevance of the particular parameter value was evaluated using sensitivity analyses (see §2.d.ii).

parameter	definition	value	reference/notes
*r*	reproductive rate, per capita	1.31	Southwood *et al*. [41]; Dye [42]
*d*	death rate, per capita	0.12	Southwood *et al*. [41]; Dye [42]
*α*	strength of density-dependent intraspecific competition, per capita	1	
*β*_i_	strength of interspecific competition per capita, relative to intraspecific competition (*α*)	varied, from 0 to 1	when *β*_i_ = 1, the strength of interspecific competition is equal to intraspecific competition. *β*_i_ is never greater than 1, as this would make species coexistence impossible [36].
*δ*_i_	strength of reproductive interference, per capita	varied, from 0 to 1	Kishi & Nakazawa [36]
*θ*	ratio of self-limiting *Ae. aegypti*: wild *Ae. aegypti*	varied, from 0 to 8	overflooding: *θ* > 1
			underflooding: *θ* < 1

### (a) Baseline models

#### (i) Kuno's model of reproductive interference

Kuno [34] developed a two species interaction model that described resource competition and reproductive interference. In this model, for the *i*th species an increase in the heterospecific population causes a rise in density-dependent resource competition, increasing mortality and causes a rise in reproductive interference, decreasing recruitment. This model can be described by the following set of differential equations:

2.1*a* $$\begin{array}{rl} \frac{\partial A(t)}{\partial t} & =r_{A}A(t)\left(\frac{A(t)}{A(t)+\delta_{A}B(t)}\right)-\alpha_{A}A(t)[A(t)+\beta_{A}B(t)]\\ & \;-d_{A}A(t) \end{array}$$

2.1*b* $$\begin{array}{rl} \frac{\partial B(t)}{\partial t} & =r_{B}B(t)\left(\frac{B(t)}{B(t)+\;\delta_{B}A(t)}\right)\;-\;\alpha_{B}B(t)[B(t)+\;\beta_{B}A(t)]\\ & \;-d_{B}B(t) \end{array}$$

where *A* represents the density of *Ae. aegypti* and *B* represents the density of *Ae. albopictus*. Parameters subscript A correspond to *Ae. aegypti*, and subscript B to *Ae. albopictus*. Recruitment is determined by *r*_i_, the reproductive rate, scaled by *δ*_i_, the decrease in reproductive success caused by reproductive interference. Density-dependent adult mortality is determined using *α*_i_, the strength of intraspecific competition, and *β*_i_, the strength of interspecific competition (relative to the intraspecific competition), while *d*_i_ is the density-independent adult mosquito mortality rate.

#### (ii) Basic self-limiting *Ae. aegypti* release

This model describes the reduction in recruitment of wild *Ae. aegypti* (denoted *A*) by the release of self-limiting *Ae. aegypti.* A proportional release policy [39] is used—thus, it is assumed that there is a stable proportion of self-limiting *Ae. aegypti* to wild *Ae. aegypti*. Furthermore, it is assumed that the self-limiting males always mate to produce non-viable offspring and are fully competitive with wild mosquitoes. This model can be described by

2.2*a* $$\begin{array}{rl} \frac{\partial A(t)}{\partial t} & =r_{A}A(t)\left(\frac{A(t)}{A(t)+\theta A(t)}\right)-\alpha_{A}A(t)[A(t)+\beta_{A}B(t)]\\ & \;-d_{A}A(t) \end{array}$$

2.2*b* $$\frac{\partial B(t)}{\partial t}=r_{B}B(t)\;-\;\alpha_{B}B(t)[B(t)+\;\beta_{B}A(t)]-d_{B}B(t)$$

where *θ* is the ratio of self-limiting *Ae. aegypti* to wild *Ae. aegypti*. All other parameters are given above (for equations (2.1*a*) and (2.1*b*)).

### (b) Self-limiting *Ae. aegypti* release with reproductive interference

These models combine Kuno's model (equations (2.1*a*) and (2.1*b*)) [34] and the basic self-limiting *Ae. aegypti* release model (equations (2.2a) and (2.2b)) to assess the impact of reproductive interference (equations (2.1*a*) and (2.1*b*)) and self-limiting *Ae. aegypti* release (equations (2.2*a*) and (2.2*b*)) on the densities of *Ae. aegypti* (denoted *A*) and *Ae. albopictus* (denoted *B*). Comparisons between the results from equations (2.3*a*) and (2.3*b*) and equations (2.4*a*) and (2.4*b*) allow the impact of reproductive interference caused by self-limiting *Ae. aegypti* and wild mosquitoes to be assessed separately.

#### (i) Self-limiting *Ae. aegypti* do not cause reproductive interference

Here, the released self-limiting *Ae. aegypti* only act to decrease the recruitment of *Ae. aegypti*:

2.3*a* $$\begin{array}{rl} \frac{\partial A(t)}{\partial t} & =r_{A}A(t)\left(\frac{A(t)}{A(t)+\;\theta A(t)+\delta_{A}B(t)}\right)-\alpha_{A}A(t)[A(t)+\beta_{A}B(t)]\\ & \;-d_{A}A(t) \end{array}$$

2.3*b* $$\begin{array}{rl} \frac{\partial B(t)}{\partial B} & =r_{B}B(t)\left(\frac{B(t)}{B(t)+\delta_{B}A(t)}\right)-\alpha_{B}B(t)[B(t)+\beta_{B}A(t)]\\ & \;-d_{B}B(t) \end{array}$$

#### (ii) Self-limiting *Ae. aegypti* cause reproductive interference

This model extends equations (2.3*a*) and (2.3*b*). Here, the released self-limiting *Ae. aegypti* reproductively interfere with *Ae. albopictus*:

2.4*a* $$\begin{array}{rl} \frac{\partial A(t)}{\partial t} & =r_{A}A(t)\left(\frac{A(t)}{A(t)+\theta A(t)+\delta_{A}B(t)}\right)-\alpha_{A}A(t)[A(t)+\beta_{A}B(t)]\\ & \;-d_{A}A(t) \end{array}$$

2.4*b* $$\begin{array}{rl} \frac{\partial B(t)}{\partial t} & =r_{B}B(t)\left(\frac{B(t)}{B(t)+\delta_{B}[A(t)+\theta A(t)]}\right)-\alpha_{B}B(t)[B(t)+\beta_{B}A(t)]\\ & \;-d_{B}B(t) \end{array}$$

This model assumes that self-limiting and wild *Ae. aegypti* equally interfere with the reproduction of *Ae. albopictus*.

### (c) Model analysis

#### (i) Zero-net-growth isoclines

Zero-net-growth isoclines were used to compare the outcomes of interspecific interaction across models. By definition, the zero-net-growth isoclines for each model were determined by solving d*A*(*t*)/d*t* = 0 and d*B*(*t*)/d*t* = 0 and taking positive solutions. This resulted in a linear (basic self-limiting *Ae. aegypti* release, equations (2.2*a*) and (2.2*b*)) or quadratic (all other models) equation for each species. Equilibria occur when the population growth rates of both species are equal zero (i.e. d*A*(*t*)/d*t* = 0 and d*B*(*t*)/d*t* = 0); where the zero-net-growth isoclines for each species cross. Equilibria were determined numerically with a multiroot function [44,45] which uses the Newton–Raphson method. Two types of equilibria are possible: exclusion of either species, or coexistence of both species. A Jacobian matrix approach was used to determine the stability of equilibrium points, where stable equilibria produce negative dominant eigenvalues and the magnitude of the eigenvalue corresponds to the stability [46].

#### (ii) Sensitivity analysis

*Population size.* We examined the impact of the strength of different parameters on the population size of each species at stable equilibria. While keeping all other parameters constant, the strength of the reproductive interference and interspecific competition experienced by each species, together with the self-limiting *Ae. aegypti* release ratio, were varied. This was conducted separately for exclusion and coexistence equilibria.

As with the isocline analysis, equilibria were determined numerically [44,45]. Under certain circumstances, the Newton–Raphson method fails to converge upon a root. For instance, when there is an inflection point at the root that is being approximated, the Newton–Raphson method often fails to converge but instead forms an oscillating sequence. Thus, for each sensitivity analysis, parameter values were selected to prevent these failures occurring. For this reason, the constant parameter values can vary between sensitivity analyses and comparisons were not made between sensitivity analyses.

*Coexistence and exclusion boundaries.* For each model with a reproductive interference term (equations (2.1*a*) and (2.1*b*), equations (2.3*a*) and (2.3*b*) and equations (2.4*a*) and (2.4*b*)), further analysis was conducted to determine the parameter space that results in stable coexistence. We examined how the strength of reproductive interference and interspecific competition influence the potential for coexistence for different self-limiting *Ae. aegypti* release ratios (no self-limiting *Ae. aegypti* release, *θ* = 0; weak underflooding, *θ* = 0.4; moderate underflooding, *θ* = 0.8; weak overflooding, *θ* = 1.2; substantial overflooding, *θ* = 8.0). We varied both the strength of reproductive interference and interspecific competition parameters from 0 (where they have no effect) to 1 (where heterospecifics are equivalent to conspecifics). In order to determine parameter values that result in stable coexistence, cubic expressions (cubic formula, equations (2.5)) were derived by substituting the solution for *Ae. aegypti* (denoted *A*) into the equation for *Ae. albopictus* (denoted *B*) and vice versa. When both cubic equations in a model have three positive solutions, there is stable coexistence—two unstable coexistence points surrounding one stable coexistence point [35]. For the coupled cubic equations to have at least three solutions, the discriminants of both equations must be greater than zero (equations (2.6)) and for all solutions to be positive, and thus biologically relevant, the coefficients have satisfy certain inequalities [35], see equations (2.7).

2.5 $$ax^{3}+bx^{2}+cx+d=0$$

2.6 $$18abcd-4b^{3}d+b^{2\;}c^{2}-4ac^{3}-27a^{2}d^{2}>0$$

2.7 $$\begin{array}{rl} & a\;\&\;c>0,\;\mathrm{and}\;\;b\;\&\;d<0,\;\mathrm{or}\\ & a\;\&\;c<0,\;\mathrm{and}\;\;b\;\&\;d>0\; \end{array}$$

All analyses (simulations, mathematical derivations and graphical analysis) were completed in R (v. 4.0.4) and Mathematica (v. 12). Code is available at (OSF) and for further details of these analyses, see electronic supplementary material [47].

## 3. Results

### (a) Isocline analysis

The addition of self-limiting *Ae. aegypti* or reproductive interference to the baseline models can alter the shape or gradient of the zero-net-growth isocline of one or both species. This changes where the two isoclines intersect and influences the number of coexistence points, their stability and the population size of each species at those points.

**Figure 2.**
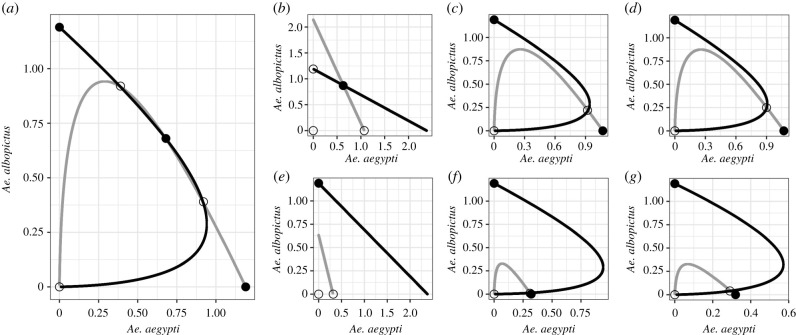
Outcomes of interspecific interaction across models. Comparison of zero-net-growth isocline plots of *Ae. aegypti* (grey) and *Ae. albopictus* (black) in an underflooding (*θ* = 0.1, row 1) and an overflooding (*θ* = 2, row 2) scenario. Isoclines of two baseline models are shown: Kuno's model of reproductive interference (equations (2.1*a*) and (2.1*b*), (*a*)) and a basic self-limiting *Ae. aegypti* release model (equations (2.2*a*) and (2.2*b*); (*b*,*e*)). The remaining plots illustrate models of self-limiting *Ae. aegypti* release with reproductive interference, where the self-limiting *Ae. aegypti* do not reproductively interfere with *Ae. albopictus* (equations (2.3*a*) and (2.3*b*); (*c*,*f*)) and where they do (equations (2.4*a*) and (2.4*b*); (*d*,*g*)). Filled circles show stable equilibria, and unfilled circles unstable equilibria. Constant parameter values are given in table 1, *β*_A_ and *β*_B_ = 0.5, and, where relevant, *δ*_A_ and *δ*_B_ = 0.15 and *θ* = 0.1 (underflooding, row 1) or 2 (overflooding, row 2).

By comparing the isoclines for Kuno's model of reproductive interference (equations (2.1*a*) and (2.1*b*); figure 2*a*) to models with reproductive interference and self-limiting *Ae. aegypti* release, we can determine the impact of releasing self-limiting *Ae. aegypti*. Where there is reproductive interference, but no release of self-limiting *Ae. aegypti* (Kuno's model, equations (2.1*a*) and (2.1*b*); figure 2*a*), *Ae. aegypti* and *Ae. albopictus* isoclines cross at three points, one stable coexistence point flanked by two unstable coexistence points (as seen in [31]). However, underflooding (figure 2*c*,*d*) and overflooding (figure 2*f*,*g*) with self-limiting *Ae. aegypti* causes vertical and horizontal compression of the *Ae. aegypti* isocline, and minor horizontal compression of the *Ae. albopictus* isocline, causing the isoclines to only cross at a single point. Therefore, following underflooding and overflooding with self-limiting *Ae. aegypti*, coexistence is destabilized and exclusion are the only stable equilibria.

The influence of reproductive interference on the stable equilibria is assessed by comparing the isoclines of the model of self-limiting *Ae. aegypti* release with no reproductive interference (equations (2.2*a*) and (2.2*b*); figure 2*b*,*e*) to the models with both self-limiting *Ae. aegypti* release and reproductive interference (equations (2.3*a*) and (2.3*b*); figure 2*c,f* and equations (2.4*a*) and (2.4*b*); figure 2*d,g*, respectively). In the absence of reproductive interference, the dynamics depend upon the release ratio of self-limiting *Ae. aegypti*; in an underflooding scenario, there is only stable coexistence (equations (2.2*a*) and (2.2*b*); figure 2*b*), however, in an overflooding scenario the only stable equilibrium is the exclusion of *Ae. aegypti* by *Ae. albopictus* (equations (2.2*a*) and (2.2*b*); figure 2*e*). Thus, where there is no reproductive interference, a high enough release ratio of self-limiting *Ae. aegypti* promotes the exclusion of *Ae. aegypti* by *Ae. albiopictus*. However, the inclusion of reproductive interference—either where self-limiting *Ae. aegypti* do not reproductively interfere with *Ae. albopictus* (equations (2.3*a*) and (2.3*b*); figure 2*c*,*f*), or where they do (equations (2.4*a*) and (2.4*b*); figure 2*d*,*g*)—results in the exclusion of *Ae. aegypti* by *Ae. albopictus* and the exclusion of *Ae. albopictus* by *Ae. aegypti* being stable equilibria. This is true in both an underflooding and overflooding scenario. Thus, in the overflooding scenario, the addition of reproductive interference stabilizes the exclusion of *Ae. albopictus* by *Ae. aegypti.* Further, in the underflooding scenario, the addition of reproductive interference destabilizes coexistence, and stabilizes the exclusion of *Ae. aegypti* by *Ae. albopictus* and the exclusion of *Ae. albopictus* by *Ae. aegypti*.

There is minimal difference between the results of the model where self-limiting *Ae. aegypti* cause reproductive interference (equations (2.4*a*) and (2.4*b*); figure 2*d*,*g*), and the model where only wild mosquitoes cause reproductive interference (equations (2.3*a*) and (2.3*b*); figure 2*c*,*f*). This suggests that the reproductive interference caused by wild *Ae. aegypti* and *Ae. albopictus* has a much greater impact on dynamics than the reproductive interference caused by self-limiting *Ae. aegypti.* This is further explored in the sensitivity analysis (see coexistence and exclusion boundaries, 3b(ii)).

### (b) Sensitivity analysis

**Figure 3.**
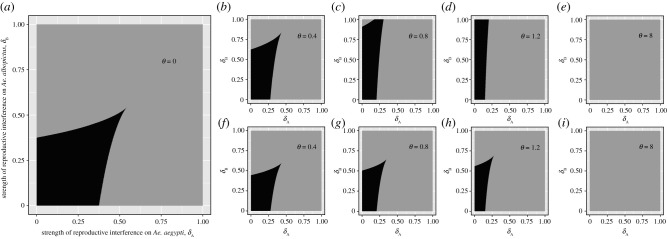
The effects of the strength of reproductive interference (*δ*_i_) on stable coexistence in all models with a reproductive interference term (equations (2.1*a*) and (2.1*b*); equations (2.3*a*) and (2.3*b*) and equations (2.4*a*) and (2.4*b*)). Parameter space where stable coexistence can occur is shaded in black. The ratio of self-limiting *Ae. aegypti* release (*θ*) is varied across plots, from no self-limiting *Ae. aegypti* release (*θ* = 0, (*a*)), to weak underflooding (*θ* = 0.4, (*b*,*f*)), moderate underflooding (*θ* = 0.8, (*c*,*g*)), weak overflooding (*θ* = 1.2, (*d*,*h*)) and substantial overflooding (*θ* = 8, (*e*,*i*)). In the first row, self-limiting *Ae. aegypti* do not reproductively interfere with *Ae. albopictus* (equations (2.3*a*) and (2.3*b*)), while in the second they do (equations (2.4*a*) and (2.4*b*)). Constant parameter values are given in table 1, and *β*_A_ and *β*_B_ = 0.1.

**Figure 4.**
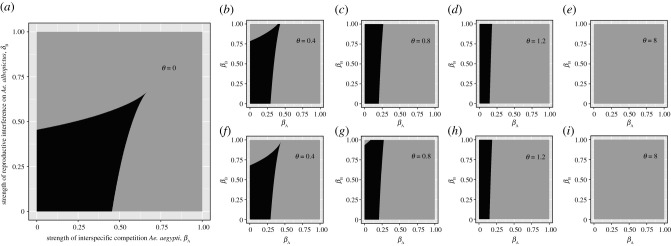
The effects of the strength of interspecific competition (*β*_i_) on stable coexistence in all models with a reproductive interference term (equations (2.1*a*) and (2.1*b*); equations (2.3*a*) and (2.3*b*) and equations (2.4*a*) and (2.4*b*)). Parameter space where stable coexistence can occur is shaded in black. As in figure 3, the ratio of self-limiting *Ae. aegypti* release (*θ*) is varied across plots, from no self-limiting *Ae. aegypti* release (*θ* = 0, (*a*)), to weak underflooding (*θ* = 0.4, (*b*,*f*)), moderate underflooding (*θ* = 0.8, (*c*,*g*)), weak overflooding (*θ* = 1.2, (*d*,*h*)) and substantial overflooding (*θ* = 8, (*e*,*i*)). In the first row, self-limiting *Ae. aegypti* do not reproductively interfere with *Ae. albopictus* (equations (2.3*a*) and (2.3*b*)), while in the second row, they do (equations (2.4*a*) and (2.4*b*)). Constant parameter values are given in table 1, and *δ*_A_ and *δ*_B_ = 0.1.

Isocline analyses are limited in that they only show stable equilibria for a certain set of parameter values. Thus, we conducted further analyses to examine the sensitivity of the population sizes of *Ae. aegypti* and *Ae. albopictus* to the ratio of self-limiting *Ae. aegypti* (*θ*; electronic supplementary material, figure S1), the strength of reproductive interference (*δ*_i_; electronic supplementary material, figure S2), and the strength of interspecific competition (*β*_i_; electronic supplementary material, figure S3). We then assessed the sensitivity of the stable coexistence of *Ae. aegypti* and *Ae. albopictus* to the strength of reproductive interference (*δ*_i_; figure 3), and interspecific competition (*β*_i_; figure 4), for different ratios of self-limiting *Ae. aegypti* (*θ*).

#### (i) Population size

We explored the impact of different parameter values on the population size of both species at all stable equilibria. The magnitude of the ratio of self-limiting *Ae. aegypti* (*θ*), reproductive interference (*δ*_i_) and interspecific competition (*β*_i_) all impact the population size of both species at coexistence. In all models with the release of self-limiting *Ae. aegypti*, as the release ratio increases, the population size of *Ae. aegypti* at coexistence decreases, and *Ae. albopictus* increases (electronic supplementary material, figure S1*a–c*). Similarly, an increase in the strength of reproductive interference or interspecific competition (electronic supplementary material figure S2 and S3, respectively) causes the population size of the species experiencing the force to decrease, and the population size of the other species to increase, where the species coexist.

Although the ratio of self-limiting *Ae. aegypti* (*θ*), the strength of reproductive interference (*δ*_i_) and strength of interspecific competition (*β*_i_) influence the population size of both species at coexistence, only the release ratio of self-limiting *Ae. aegypti* (*θ*) has any impact on population size when there is exclusion (electronic supplementary material figure S1*d–f*). When there is reproductive interference, as the release ratio of self-limiting *Ae. aegypti* (*θ*) increases, there is a decrease in the population size of *Ae. aegypti* when it excludes *Ae. albopictus* (electronic supplementary material figure S1*e*,*f*). These results are sensical: following exclusion of a species, interspecific interactions (through resource competition and reproductive interference) will not occur, and thus will not influence population density. However, following the exclusion of *Ae. albopictus*, self-limiting *Ae. aegypti* will still suppress the population size of *Ae. aegypti*. In the absence of reproductive interference, *Ae. aegypti* cannot stably exclude *Ae. albopictus* (electronic supplementary material figure S1*d*). For all self-limiting insect release models, the population size of *Ae. albopictus*, when it excludes *Ae. aegypti*, is unaffected by the self-limiting *Ae. aegypti* release ratio (electronic supplementary material figure S1*d–f*). Again, this is sensical, as there will be no self-limiting *Ae. aegypti* present following the exclusion of *Ae. aegypti*.

#### (ii) Coexistence and exclusion boundaries

For all models with the reproductive interference term, analyses explored the ranges of strengths of reproductive interference (*δ*_i_, figure 3) and interspecific competition (*β*_i_, figure 4) that resulted in stable coexistence, for different ratios of self-limiting *Ae. aegypti* (*θ*). Similar patterns are observed for both parameters, in Kuno's model of reproductive interference (equations (2.1*a*) and (2.1*b*)) and the models including self-limiting *Ae. aegypti* release (equations (2.3*a*) and (2.3*b*) and equations (2.4*a*) and (2.4*b*)).

In Kuno's model of reproductive interference model (equations (2.1*a*) and (2.1*b*)), coexistence regions are biased towards the left corner, where there is low reproductive interference (figure 3*a*), or low interspecific competition (figure 4*a*) experienced by both species. In models with self-limiting *Ae. aegypti* release, an increase in the release ratio (*θ*) causes the stable coexistence parameter space to decrease in area, and change shape. As the release ratio (*θ*) increases, coexistence can occur when *Ae. albopictus* suffers increasingly high reproductive interference (figure 3) or interspecific competition (figure 4), and *Ae. aegypti* suffers increasingly low reproductive interference (figure 3) or interspecific competition (figure 4).

When self-limiting *Ae. aegypti* do not reproductively interfere with *Ae. albopictus* (figures 3*b–e* and 4*b–e*), coexistence can occur when *Ae. albopictus* suffers greater reproductive interference and interspecific competition, than when *Ae. aegypti* do cause reproductive interference (figures 3*f–i* and 4*f–i*). In the substantial overflooding situation, the self-limiting insect release ratio is high enough (*θ* = 8) that coexistence cannot occur for any values for reproductive interference (figure 3*e*,*i*) or interspecific competition (figure 4*e*,*i*)—thus, there is only exclusion.

## 4. Discussion

This work is the first to investigate the combined role of reproductive interference and self-limiting insect releases on the coexistence of closely related disease vectors, *Ae. aegypti* and *Ae. albopictus*. It is well established, both theoretically [47–50] and through a limited set of field trials [51,52], that the release of self-limiting mosquitoes can be used to suppress the population size of *Ae. aegypti*, and therefore, potentially, reduce the substantial associated public health burden.

*Ae. aegypti* shares a similar ecological niche with *Ae. albopictus*, they compete for resources [24–26] and interfere with each other's mating attempts [36–38]. Previous studies have separately modelled the impact of reproductive interference [33] and self-limiting *Ae. aegypti* release [21] upon the coexistence of *Ae. aegypti* and *Ae. albopictus*. However, as highlighted [35], the combined effect has not been investigated. We addressed this by examining the interactions between *Ae. aegypti* and *Ae. albopictus* where there is reproductive interference, and self-limiting *Ae. aegypti* release. We explored the potential outcomes following the release of self-limiting *Ae. aegypti*, where *Ae. aegypti* and *Ae. albopictus* coexist. This work remains highly relevant as *Ae. albopictus* and *Ae. aegypti* are undergoing range expansion, making it increasingly likely that these important vectors of disease will come into contact [30]. Our results show that the ratio of self-limiting *Ae. aegypti* and the strength of reproductive interference can act concomitantly to determine whether coexistence is maintained, or exclusion occurs. Self-limiting *Ae. aegypti* releases and reproductive interference also affect the population size of one or both species. Therefore, in locations where the distributions of *Ae. aegypti* and *Ae. albopictus* overlap, both the behavioural ecological and population ecological effects of self-limiting releases have important consequences for the efficacy of vector control programmes.

To investigate the release of self-limiting *Ae. aegypti*, we used a proportional release policy [40], where, at each time point, the number of self-limiting *Ae. aegypti* is proportional to the number of wild *Ae. aegypti* [40]. To conduct proportional releases in the field requires constant monitoring of mosquito populations. There are well established techniques to monitor *Aedes* populations, and entomological surveys are necessary following self-limiting insect release in order to monitor efficacy [19]. The proportional release policy has been investigated in previous theoretical studies on self-limiting control [39,53,54]; under this release policy, fewer self-limiting mosquitoes need to be released to eradicate the target organism than in a constant release scenario (where the same number of self-limiting mosquitoes are released at each time step) [39]. As such, there could be the economic benefit of using proportional release policies, especially in areas where public health monitoring of mosquitoes is sufficient to estimate mosquito density, so no additional monitoring costs accrue. Economic benefits are an important consideration: cost-effectiveness has been highlighted as a technical aspect of self-limiting insect releases that requires further investigation [19]. One approach could be to compare the costs of different release policies to understand the implications of self-limiting releases on wider aspects of biodiversity.

### (a) Population size

We examined the impact of increasing the ratio of self-limiting *Ae. aegypti* on the population sizes of *Ae. aegypti* and *Ae. albopictus*. Self-limiting *Ae. aegypti* mate with their wild counterparts to produce non-viable offspring: thus, these self-limiting insects suppress the population density of *Ae. aegypti* by decreasing recruitment. This is shown from our analyses, where increasing the ratio of self-limiting *Ae. aegypti* decreases the population density of *Ae. aegypti* when there is coexistence, or where *Ae. aegypti* excludes *Ae. albopictus*. As there are fewer *Ae. aegypti* to compete with *Ae. albopictus* for resources or interfere with their mating, the population size of *Ae. albopictus* increases with the self-limiting *Ae. aegypti* release ratio when there is coexistence. This result holds when self-limiting *Ae. aegypti* reproductively interfere with *Ae. albopictus*, and when they do not cause reproductive interference: thus, this outcome is governed by the decrease in the wild *Ae. aegypti* population size, rather than any additional increase in reproductive interference by self-limiting *Ae. aegypti*.

Following the release of self-limiting *Ae. aegypti*, where *Ae. albopictus* persists, it will increase in population size; either by excluding *Ae. aegypti* and reaching carrying capacity, or by increasing its population size in coexistence with *Ae. aegypti*. This is an important consideration: as highlighted by Bargielowski *et al.*, *Ae. albopictus* is the principal vector of dengue in regions where *Ae. aegypti* is rare or uncommon (e.g. in China [55–57], Bangladesh [58] and South India [59,60]), and in regions of Africa native to *Ae. aegypti* that *Ae. albopictus* has recently colonized [61]. Therefore, in some regions, the decrease in public health burden caused by lowering the *Ae. aegypti* population may be lessened or compensated for by the associated increase in the *Ae. albopictus* population size. This highlights a limitation of using species specific pest control techniques [18]. In situations where both species coexist and are significant disease vectors, the release of both self-limiting *Ae. aegypti* and *Ae. albopictus* may be a more appropriate course of action [19].

Furthermore, our results show that the strength of reproductive interference and interspecific competition affects the population densities of both species at coexistence. An increase in the strength of reproductive interference or interspecific competition reduces the population size of the species experiencing the behavioural (reproductive interference) or ecological (interspecific competition) effects. For the former, this is due to fewer successful matings, reducing recruitment, and for the latter, more competition with heterospecifics for resources, increasing the number of deaths. The subsequent reduction in population size of the focal species allows the other species to increase in population density, as there are fewer heterospecifics to compete with for resources, or to interfere with their mating.

However, previous work has shown *Ae. aegypti* may develop some resistance to reproductive interference. Bargielowski *et al.* [37] showed that female *Ae. aegypti* from populations in allopatry with *Ae. albopictus* mis-mate more frequently than those from populations with a history of sympatry; suggesting that upon contact between *Ae. aegypti* and *Ae. albopictus*, *Ae. aegypti* are selected for their ability to evade reproductive interference by *Ae. albopictus.* Thus, where *Ae. aegypti* and *Ae. albopictus* have come into contact more recently, *Ae. aegypti* may experience stronger reproductive interference. Our results suggest this will cause a lower population size of *Ae. aegypti—*thus, recent contact with *Ae. albopictus* means fewer self-limiting *Ae. aegypti* need to be released to have the equivalent impact upon the *Ae. aegypti* population size. Therefore, these areas could be targeted at a lower economic burden. This is particularly relevant in regions where *Ae. albopictus* is not a disease vector.

### (b) Coexistence and exclusion boundaries

Our results show that the ratio of self-limiting *Ae. aegypti* released determines whether coexistence is possible. A high enough release ratio of *Ae. aegypti* destabilizes coexistence across all strengths of reproductive interference and interspecific competition*—*thus, the only stable outcome is exclusion. However, at lower release ratios, coexistence is still possible. Here, the ratio of self-limiting *Ae. aegypti*, and whether the self-limiting *Ae. aegypti* cause reproductive interference, determines the strength of the reproductive interference and resource competition that can be experienced by *Ae. aegypti* and *Ae. albopictus*, and coexistence maintained.

When self-limiting *Ae. aegypti* reproductively interfere with *Ae. albopictus*, coexistence can occur when each individual *Ae. aegypti* causes weaker reproductive interference and interspecific competition, than when only wild *Ae. aegypti* reproductively interfere with *Ae. albopictus*. This is because, there are additional individuals (the self-limiting *Ae. aegypti*) that reproductively interfere with *Ae. albopictus*. This increases the total reproductive interference experienced by the *Ae. albopictus* population, for the same strength of reproductive interference imposed by each individual *Ae. aegypti*.

As discussed, an increase in the ratio of self-limiting *Ae. aegypti* causes the population size of *Ae. aegypti* to decrease, meaning there are fewer *Ae aegypti* to interfere with the mating of *Ae. albopictus* or compete with them for resources. Thus, as the release ratio increases, each individual *Ae. aegypti* can reproductively interfere and compete for resources increasingly strongly with *Ae. albopictus*, without changing the total ecological effects experienced by the *Ae. albopictus* population. As the population size of *Ae. albopictus* increases with the release ratio of self-limiting *Ae. aegypti*, the reverse occurs to the per capita reproductive interference and competition for resources caused by *Ae. albopictus*. For these reasons, up to a moderate release ratio of *Ae. aegypti*, our results show that as the ratio of self-limiting *Ae. aegypti* increases, coexistence can occur when the reproductive interference and resource competition suffered by *Ae. albopictus* is increasingly high and that suffered by *Ae. aegypti* is increasingly low.

However, meta-analyses suggest that *Ae. albopictus* is more likely to experience weaker reproductive interference, and interspecific resource competition than *Ae. aegypti* [30,42]. A meta-analysis by Juliano [42] showed *Ae. albopictus* has a competitive advantage over *Ae. aegypti* when there is low food quality, and high food quality results in competitive equivalence. Furthermore, *Ae. aegypti* is more likely to mis-mate than *Ae. albopictus* [37,38] and following mis-mating, female *Ae. aegypti* can become refractory to further mating [36]. This asymmetry is likely to be due to the greater species recognition abilities of *Ae. albopictus* [31,38]. This suggests that in the wild, as the release ratio of self-limiting *Ae. aeypti* increases, coexistence will only occur where both species experience an increasingly low strength of reproductive interference and interspecific resource competition, although coexistence is theoretically possible when *Ae. albopictus* suffers more than *Ae. aegypti*.

It is plausible that there are regions where the reproductive interference and resource competition experienced by *Ae. aegypti* is sufficiently weak for coexistence to occur, even when self-limiting *Ae. aegypti* are released. For instance, coexistence could be facilitated in areas with high food quality, where the competitive advantage of *Ae. albopictus* is reduced, resulting in lower interspecific competition [42]. Furthermore, *Ae. aegypti* may experience reproductive interference that is weak enough to allow coexistence following the release of self-limiting *Ae. aegypti—*for instance in a coexistence region in Brazil, *Ae. albopictus* only weakly reproductively interferes with *Ae. aegypti* [43]. Equally, as discussed, in regions where *Ae. aegypti* initially experiences strong reproductive interference, this species could develop resistance to reproductive interference prior to exclusion [37]. Additionally, in the wild, habitat partitioning in time or space could allow reproductive interference to be avoided and coexistence to be maintained [62]. Thus, the release of self-limiting *Ae. aegypti* could initially destabilize coexistence, but subsequent development of resistance to mis-mating in *Ae. aegypti*, or habitat partitioning, could recover stable coexistence.

## 5. Conclusion

Previous models have examined the impact of reproductive interference [33–35] and self-limiting insect release [21] on the population dynamics between the vectors *Ae. aegypti* and *Ae. albopictus*. However, we are the first to addresses the combined impact of self-limiting insect release and reproductive interference upon *Ae. aegypti* and *Ae. albopictus* coexistence. Our results show that the strength of reproductive interference and the ratio of self-limiting *Ae. aegypti* are important factors that act together to determine the population size of both *Ae. aegypti* and *Ae. albopictus* and whether coexistence can occur. This highlights the importance of including behavioural ecological processes, such as reproductive interference, into population dynamic frameworks to evaluate the efficacy of vector control. Future work could focus on stochastic and spatial aspects of coexistence following self-limiting releases. Furthermore, linking these outcomes to vector-borne disease epidemiology would allow the effects of behavioural traits (such as reproductive interference) on disease spread and the public health implications to be evaluated.

## Data accessibility

Code and electronic supplementary material can be found at: https://osf.io/uqwfr/?view_only=d3190b8c17704af884e2dcd2bed0dd3e [45].
